# Cytokines profiling by multiplex analysis in experimental arthritis: which pathophysiological relevance for articular versus systemic mediators?

**DOI:** 10.1186/ar3774

**Published:** 2012-03-13

**Authors:** Joseph Paquet, Jean-Christophe Goebel, Camille Delaunay, Astrid Pinzano, Laurent Grossin, Christel Cournil-Henrionnet, Pierre Gillet, Patrick Netter, Jean-Yves Jouzeau, David Moulin

**Affiliations:** 1 Physiopathologie, Pharmacologie et Ingénierie Articulaire - PPIA-UMR 7561 CNRS UHP, Université de Lorraine, Faculté de Médecine, BP 184, 54505 Vandoeuvre Les Nancy, France; 2 Anatomie et Cytologie Pathologiques - CHU Nancy Brabois, 54505 Vandoeuvre Les Nancy, France

## Abstract

**Introduction:**

We have taken advantage of the large screening capacity of a multiplex immunoassay to better define the respective contribution of articular *versus *systemic cytokines in experimental arthritis.

**Methods:**

We performed a follow up (from 7 hours to 14 days) multiplex analysis of 24 cytokines in synovial fluid and sera of rats developing Antigen-Induced Arthritis (AIA) and confronted their protein level changes with molecular, biochemical, histological and clinical events occurring in the course of the disease.

**Results:**

The time-scheduled findings in arthritic joints correlated with time-dependent changes of cytokine amounts in joint effusions but not with their blood levels. From seven hours after sensitization, high levels of chemokines (MCP-1, MIP1α, GRO/KC, RANTES, eotaxin) were found in synovial fluid of arthritic knees whereas perivascular infiltration occurred in the synovium; local release of inflammatory cytokines (IFNγ, IL-1β, IL-6) preceded the spreading of inflammation and resulted in progressive degradation of cartilage and bone. Finally a local overexpression of several cytokines/adipocytokines poorly described in arthritis (IL-13, IL-18, leptin) was observed.

**Conclusions:**

Distinct panels of cytokines were found in arthritic fluid during AIA, and the expected effect of mediators correlated well with changes occurring in joint tissues. Moreover, multiplex analysis could be helpful to identify new pathogenic mediators and to elucidate the mechanisms supporting the efficacy of putative targeted therapies.

## Introduction

Rheumatoid arthritis (RA) is a chronic inflammatory autoimmune disorder [1] characterized by infiltration of neutrophils and lymphocytes into the synovial tissue and joint fluid [2,3], leading to secondary cartilage and bone destruction [4]. Several mediators, including proinflammatory and immunomodulatory cytokines, growth factors, and chemotactic cytokines (chemokines), have been implicated in the inflammatory process of RA. Although the cytokine network suggests that mediators can be categorized depending on their primary or secondary role in the disease process [5], questions about the fine tuning of cytokine expression during autoimmune arthritis remain. Cytokines such as interleukin-1-beta (IL-1β), tumor necrosis factor-alpha (TNF-α), and interleukin-6 (IL-6) [6,7] have been shown to display potent proinflammatory actions and to contribute to the pathogenesis of RA [8] or experimental arthritis, particularly to cartilage and bone damages [9]. Thus, targeted therapies against these cytokines were shown to be relevant in murine and rat models of RA [10,11] before being shown to be efficient therapies in the clinics [12]. Anti-TNFα therapies are now widely used [13] and their clinical benefits are well recognized despite an increased risk of infectious disease such as tuberculosis [14]. However, regardless of the therapy used, some patients treated with anticytokine biotherapies remain refractory or become non-responders to the treatment. Therefore, there is a need to use combined therapies or to search for new therapeutic strategy aiming to control additional mediators or both [15].

Antigen-induced arthritis (AIA), a severe monoarticular chronic arthritis induced by intra-articular administration of methylated bovine serum albumin (mBSA) in sensitized animals, is an immune-mediated joint inflammation reproducing some histopathological findings of RA, such as infiltration of the synovial membrane by CD4^+ ^T cells and macrophages and a disease course with discernable phases [16,17]. The kinetics and pathogenic roles of cytokines and chemokines have not been extensively investigated in AIA, and analysis of their local expression could help to elucidate the mechanisms supporting arthritis [18]. Earlier studies of the expression of mediators in this model were carried out on joint homogenates [19,20] mainly because the available amounts of tissue samples were too small to allow an extensive analysis. In addition, these data considered the mRNA levels mainly and were not able to distinguish mature cytokines from their precursors, thus overestimating the amount of several active mediators with a possible pathogenic role. When studies focused on the cytokine levels in joint fluid of arthritic rodents, a limited number of mediators were addressed [19,21] or assays were restricted to cytokines, such as TNF-α, IL-1β, or IL-6, that have a well-known pathogenic role [22].

In the present study, we investigated the kinetics of 24 cytokines in rats developing AIA using a multiplex immunoassay that allowed a highly sensitive biological follow-up of multiple mediators from a limited amount of biological sample [15]. Levels of mediators were checked concomitantly in the knee joint - that is, the synovial fluid (SF) - and the bloodstream to establish the sequence and display of cytokine activation in RA-like conditions and to correlate the time-dependent changes of mediators with clinical and histological hallmarks of arthritis. We observed induction of IL-1β, IL-6 and IL-17, cytokines already described as key players in the arthritis process and clinically used as therapeutic targets. Interestingly, we also found expression of mediators that, to date, have no known involvement in this pathology. Indeed, the chemokines eotaxin and growth-related oncogene/keratinocyte chemoattractant (GRO/KC) and the T helper 2 (Th2)-associated cytokines IL-13 and IL-9 were highly induced in arthritic SFs and represent new potential targets for RA treatment.

## Materials and methods

### Animals

All experiments were carried out in barrier-maintained male Wistar Han rats (150 to 175 g on day 0, or D_0_) purchased from Charles River Laboratories (L'Arbresle, France). Animals were allowed to acclimatize for at least 1 week after their arrival in our facility. Animals were housed in groups of five in solid-bottomed plastic cages with access to tap water and standard rodent pelleted chow (A04; Scientific Animal Food & Engineering, Villemoisson-sur-orge, France) *ad libitum*. Room temperature was set at 23 ± 1°C, and animals were subjected to a 12-hour light cycle (with lights on from 6 a.m. to 6 p.m.). All experiments were performed in accordance with national animal care guidelines and were preapproved by a local ethics committee. Arthritis induction, blood sampling, and necropsy were performed under general anesthesia by using volatile anesthetics (AErrane™; Baxter SA, Maurepas, France).

### Induction of antigen arthritis

Animals were immunized 21 and 14 days before the antigenic challenge by a subcutaneous flank injection of 250 μL of a suspension, which contained 0.5 g of methylated bovine serum albumin (mBSA) (Sigma-Aldrich, Deisenhofen, Germany) and which was resuspended in 125 μL of saline and emulsified with 125 μL of complete Freund's adjuvant (2 mg/mL *Mycobacterium tuberculosis*; Difco Laboratories Inc., now part of Becton Dickinson and Company, Franklin Lakes, NJ, USA). On D_0_, AIA was induced by a single intra-articular injection of 0.5 mg of mBSA (50 μL of 10 mg/mL mBSA dissolved in 0.9% NaCl) into the right knee joint; the contralateral knee received 50 μL of 0.9% NaCl. Control rats were immunized with the same protocol but received an intra-articular injection of 50 μL of 0.9% NaCl in both knees on D_0_.

### Scoring of arthritis

The disease course was monitored by the repeated assessment of knee joint width by using a caliper (Kroeplin Längenmesstechnik, Schlüchtern, Germany). Raw data were used to estimate joint circumference by using the geometric formula of ellipse circumference, 2πX to the power of √ (*a*^2 ^*+ B*^2^), in which *a *is knee height and *B *is knee breadth. The results were representative of joint swelling and expressed (in millimeters) as the difference between the joint size at a given day compared with that measured just before arthritis induction (D_0_).

### Weight-bearing assessment protocol

Hind-limb weight bearing was determined by using an incapacitation tester (Linton Instrumentation, Norfolk, UK) consisting of a dual-channel weight averager. Weight distribution was measured between sensitized (intra-articular injection of mBSA) and contralateral (saline-injected) hind limbs and was used as an index of joint discomfort in the arthritic knee. Rats were placed carefully in an angled Plexiglas chamber positioned so that each hindpaw rested on a separate force plate. Care was taken to ensure that the weight of the animal was directed onto the force plates and not dissipated through the walls of the chamber. The force exerted by each hind limb (measured in grams) was averaged over a 5-second period. Each data point is the mean of three readings. The percentage of weight distributed onto the sensitized (arthritic) hind limb was calculated by the following equation: [weight on ipsilateral hind limb/(weight on ispsilateral + weight on contralateral)] × 100 [23]. The weight-bearing distribution of arthritic rats was compared with that of control rats (injected bilaterally with saline into the knee joints).

### Histological examination

Knee joints were collected at necropsy, fixed immediately for 24 hours in 4% paraformaldehyde, decalcified in rapid bone decalcifier (RDO; Apex Engineering, Plainfield, IL, USA) for 6 hours at room temperature, and further fixed in 4% paraformaldehyde before embedding in paraffin. Sections (5 μm thick) were rehydrated in a graded ethanol series and stained with either hematoxylin/eosin/safran, safranin O-fast green, or May-Grünwald Giemsa. The histological characteristics of articular cartilage, bone, and peri-articular soft tissues were scored independently by two observers who were blind to the samples. Cartilage degradation was graded from 0 to 3, where 0 = fully stained cartilage, 1 = loss of proteoglycan staining in the superficial layer, 2 = complete loss of proteoglycan staining, and 3 = complete loss of cartilage. The following morphological criteria were used for bone erosion: 0 = normal, 1 = mild loss of cortical bone at few sites, 2 = moderate loss of cortical and trabecular bone, and 3 = marked loss of bone at many sites. Synovium from the knee joint was graded by using a scoring technique adapted from Rooney and colleagues [24]. Briefly, samples were evaluated on a scale from 0 to 4 (0 = normal and 4 = major changes) for hyperplasia of synovial fibroblasts (depth of lining layer), fibrosis (percentage of replacement of loose connective tissue), angiogenesis (number of proliferating blood vessels), perivascular infiltrates of lymphocytes (percentage of vessels surrounded by lymphocytes), and tissue infiltration by lymphocytes (size of aggregates and percentage of infiltrating cells).

### Assessment of cytokine expression

#### Biological fluid sampling

Blood (300 μL) was collected by sampling of the tail veins at seven time points: D_0_, 7 hours (H_7_), D_1_, D_2_, D_3_, D_7_, and D_14_. As we observed no significant difference between cytokine pattern measured in plasma versus serum samples in a preliminary set of experiments (not presented in this paper), we opted for cytokine analysis in serum as this method is widely accepted in cytokine pattern determination in the context of diagnosis or prognosis of RA or both [25,26]. After clotting for 1 hour at room temperature, samples were centrifuged for 10 minutes at 3,000*g *at room temperature. Obviously hemolyzed samples were discarded as they resulted in a high aggregation of beads. Serum was collected and frozen at -80°C until analysis.

Joint fluid sampling was carried out after killing of animals at corresponding times. Briefly, the patellar ligament was cut and the articular cavity was incised perpendicularly to the patella. The SF was then collected by impregnation of standardized small pieces (4 mm^2^) of filter paper (Schleicher & Schuell GmbH, Dassel, Germany). This technique was chosen because of the inability to aspirate joint fluid from rodent joints, especially saline-injected knees. The saturation of filter pieces with SF allows the circumvention of the variation in a sample volume and was applied successfully to the monitoring of nitric oxide release in rat arthritis [27]. To prevent any proteolytic cleavage of cytokines in arthritic fluids, these paper pieces were left for 12 hours at 4°C in 150 μL of PBS containing a cocktail of protease inhibitors (Complete Mini™; Roche, Basel, Switzerland, Roche reference number 11 836 153 001, one tablet for 10 mL). After initial and final agitations for 30 seconds on a mechanic stirrer, the 'joint-derived' eluates (referred to as SF) were frozen at -80°C until processing. Samples were assessed with a hemoglobin detection reagent strip to exclude any blood contamination. Briefly, 10 μL of diluted samples (1/10 in PBS) was dropped on a Hemastix^® ^reagent strip (limit of detection of 0.015 to 0.062 mg/dL, which is approximately equivalent to 5 to 20 red blood cells per microliter). Positive samples were excluded from multiplex analysis.

### Multiplex immunoassay

Levels of 24 cytokines - IL-1α, IL-1β, IL-2, IL-4, IL-5, IL-6, IL-9, IL-10, IL-12p(70), IL-13, IL-17, IL-18, leptin, GRO/KC, TNFα, interferon gamma (IFNγ), GM-CSF, RANTES, MCP-1, macrophage inflammatory protein-1-alpha (MIP-1α), G-CSF, IP-10, eotaxin, and VEGF - were determined in both SF (sensitized and contralateral) and serum at each time point by means of a Milliplex™ MAP kit (Millipore, Billerica, MA, USA). Millipore multiscreen 96-well filter plates for multiplex cytokine kits were used. Rat cytokine standards were diluted in a 'serum matrix' solution (optimized protein concentration) for serum sample determination and in PBS for SF measurements. For each time point, data were obtained from five rats. Each animal sample was run in triplicate in accordance with the protocol of the manufacturer [15], and the final results presented here are representative of three independent experiments. Data were collected by using Luminex-100 software version 1.7 (Luminex, Austin, TX, USA), and analysis was performed with the MasterPlex QT 1.0 system (MiraiBio, Alameda, CA, USA). A five-parameter regression formula was used to calculate the sample concentrations from the standard curves. Data were analyzed by using either 5- or 4-parameter logistic or spline curve-fitting method as recommended by the manufacturer. Type of curve-fitting method was chosen for each cytokine with respect to the lowest residual variance (< 5%).

### mRNA levels of selected mediators in synovium

#### Tissue sampling and preparation

Articular synovium, cartilage of patella were collected aseptically at necropsy and immediately frozen. Frozen tissues, kept at -80°C before extraction, were homogenized immediately after thawing by using a dispersing system and mixed with 350 μL of RLT buffer added with β-mercapto-ethanol. Samples were then passed on Qiashredder columns (RNeasy kit; Qiagen, Courtaboeuf, France), and mRNAs were extracted with the RNeasy kit in accordance with manufacturer recommendations.

#### Real-time reverse transcriptase-polymerase chain reaction

mRNA (0.5 μg) of each sample was then reverse-transcribed for 90 minutes at 37°C with 200 U of Moloney murine leukemia virus reverse transcriptase (Invitrogen Corporation, Carlsbad, CA, USA) and oligo(dT) primers (Eurogentec, Liège, Belgium) in accordance with the recommendations of the suppliers. Expressions of MCP-1 (chemokine) and IL-1β, IL-6, and TNF-α (proinflammatory cytokines) and VEGF (growth factor) were quantified by real-time polymerase chain reaction (PCR) with the Lightcycler^® ^(Roche) technology and the SYBR green master mix system^® ^(Qiagen). After amplification, a melting curve was constructed to determine the melting temperature of each PCR product. The mRNA levels of each gene of interest and of the ribosomal protein S29, chosen as a housekeeping gene, were determined in parallel for each sample. Results are expressed with the delta delta threshold cycle (Ct) method. The gene-specific primer pairs used were as follows: MCP-1, forward 5'-CAGATCTCTCTTCCTCCACCACTAT-3', reverse 5'-GCATTAACTGCATCTGGCTGAGACAGC-3'; IL-1β, forward 5'- CTTCCCCAGGACATGCTAGG-3', reverse 5'-CAAAGGCTTCCCCTGGAGAC-3'; IL-6, forward 5'-CCGGAGAGGAGACTTCACAG-3', reverse 5'-ACAGTGCATCATCGCTGTTC-3'; TNF-α, forward 5'-AGCCCTGGTATGAGCCCATGTA-3', reverse 5'- CCGGACTCCGTGATGTCTAAGT-3'; and VEGF, forward 5'- CACATCTGCAAGTACGTTCGTTTA-3', reverse 5'- CAGAGCGGAGAAAGCATTTGTT-3'.

### Assessment of proteoglycan metabolism in patellar cartilage

Proteoglycan synthesis was studied by an *ex vivo *incorporation of Na_2_^35^SO_4 _into patellar cartilage. At necropsy, patellas were collected aseptically, dissected from peri-articular tissues, and then pulsed for 3 hours at 37°C in a 5% CO_2 _atmosphere with 0.6 μCi/mL Na_2_^35^SO_4 _(Amersham, Les Ulis, France) in RPMI-Hepes 1640 medium supplemented with 2 mM L-glutamine, 100 IU/mL of penicillin, and 100 μg/mL of streptomycin (Life Technologies, Cergy-Pontoise, France). After five washings in saline, patellas were fixed overnight in 0.5% cetylpyridinium chloride (Sigma-Aldrich, Saint Quentin-Fallavier, France) in 10% (vol/vol) phosphate-buffered formalin and then decalcified in 5% (vol/vol) formic acid for 7 hours at room temperature. Biopsy punches, 2 mm in diameter, were taken from the central part of the patellas before dissolution overnight in Solvable (Packard, Rungis, France). ^35^S-proteoglycan content was measured by liquid scintillation counting (Hionic Fluor; Packard), data are expressed as the percentage of variation from healthy controls, and a negative value represents a decrease of proteoglycan synthesis [28].

### Statistical analysis

All results, except in Figure 2 (fold changes), are expressed as mean ± standard error of the mean. All analyses and figure editing were carried out by using GraphPad Prism (release 4; GraphPad Software, Inc., San Diego, CA, USA). The Student *t *test was used to compare a batch with its own control and analysis of variance with *post hoc *Bonferroni when required (that is, with groups of at least three).

## Results

### Clinical features of antigen-induced arthritis

**Figure 1:**
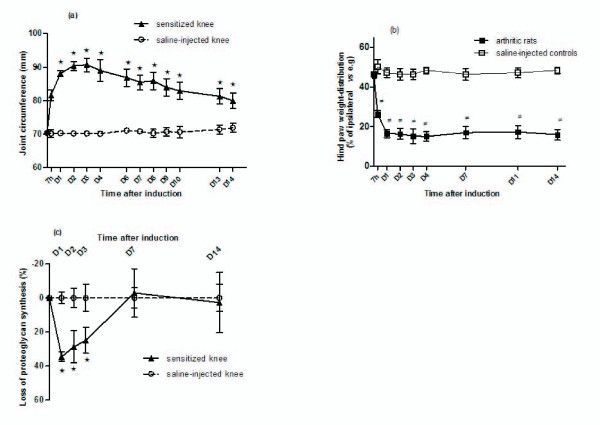
**Time course of arthritis-related changes in rats with antigen-induced-arthritis**. **(a) **Joint circumference of rat knees is estimated from joint width (as described in Materials and methods). **(b) **Hind-paw weight distribution of arthritic rats is compared with that of saline-injected controls. Data are expressed as percentage of weight distribution onto the right knee (sensitized with mBSA or saline-injected for control rats). **(c) **Proteoglycan synthesis in patellar cartilage is measured by Na_2_^35^SO_4 _incorporation. Data are expressed as the percentage of change in ^35^sulfate incorporation in the central part of the patella. Data are mean ± standard error of the mean from 28 rats (a,b) or five samples (c). *P < 0.05 sensitized versus saline-injected knee. ^≠^*P *< 0.05 arthritic versus saline-injected rats. mBSA, methylated bovine serum albumin.

All animals developed signs of arthritis from 7 hours after intra-articular injection of mBSA. A progressive joint swelling was observed in sensitized knees and peaked between D_2 _and D_4 _and then decreased slowly from D_5 _to D_14 _(Figure 1a). At the same time, a significant loss in body-weight gain was observed in arthritic rats compared with controls and reached 20.5% ± 3.5% at D_14 _(data not shown).

### Hind-limb weight distribution

Rats injected bilaterally with saline distributed their body weight between both hind limbs, as shown by an average weight distribution of 50% ± 4% between the two legs (Figure 1b). In arthritic rats, a shift in weight distribution occurred toward the saline-injected contralateral limb from 7 hours after mBSA injection. A maximal level of incapacitation was observed by D_1 _with rats placing only 16% ± 2% of their body weight on the sensitized leg (Figure 1b). Thereafter, joint discomfort remained stable until D_14_.

### Proteoglycan synthesis in patellar cartilage

In arthritic rats, a significant depletion of proteoglycan synthesis was observed from 1 to 3 days after the antigenic challenge (Figure 1c). Proteoglycan loss varied between 35% ± 2% and 29% ± 5% at these times. At a later time, proteoglycan synthesis returned to a normal level in mBSA-injected knees (Figure 1c).

### Mediators mRNA expression in joint tissues

To check for the relevance of proteins found in the SF to molecular changes occurring in inflamed synovium, mRNA expression profiles of selected cytokines were analyzed in synovial membranes of arthritic rats (Table 1). These cytokines were representative of different functional classes: one chemokine (MCP-1), three proinflammatory cytokines (IL-1β, IL-6, and TNFα), and one growth factor (VEGF). In arthritic synovium, a significant increase in mRNA levels of MCP1, IL-1β, IL-6, TNFα, and VEGF was observed as early as 7 hours after the antigenic challenge, but the time course of gene expression varied with the mediator. MCP-1 mRNA level peaked (around 14-fold) at 7 hours and decreased quickly between D_1 _and D_3 _and then slowly until D_14_. IL-1β, TNFα, and VEGF displayed a comparable profile with a maximal induction by D_1 _(50-, 12-, and 20-fold increase, respectively) and a return to normal level from D_2 _for TNFα and after D_3 _for the others. In contrast, IL-6 mRNA level peaked by D_2 _(around 158-fold) and remained significantly elevated until D_14 _(29-fold increase). None of the mediators displayed any significant change in mRNA level in the synovium of the saline-injected (contralateral) knee.

**Table 1:** Changes in cytokine mRNA level (fold change relative to day 0) in arthritic synovium during antigen-induced arthritis

Time point	MCP-1		IL-1β		TNF-α		VEGF		IL-6	
	Sensitized	Saline-injected	Sensitized	Saline-injected	Sensitized	Saline-injected	Sensitized	Saline-injected	Sensitized	Saline-injected
Day 0	1.0 ± 0.2	1.0 ± 0.1	1.0 ± 0.1	1.0 ± 0.2	1.0 ± 0.4	1.0 ± 0.5	1.0 ± 0.1	1.0 ± 0.2	1.0 ± 0.2	1.0 ± 0.1
7 hours	13.9^a ^± 5.4	1.5 ± 0.6	30.2^a ^± 4.7	1.1 ± 0.2	5.13^a ^± 2.4	1.2 ± 0.5	3.8^a ^± 0.5	1.1 ± 0.2	35.0^a ^± 8.5	2.1 ± 1.0
Day 1	5.9^a ^± 1.9	1.1 ± 0.2	49.6^a ^± 29.4	1.0 ± 0.2	11.7^a ^± 5.2	1.1 ± 0.6	20.0^a ^± 12.2	1.1 ± 0.3	12.2^a ^± 2.6	1.2 ± 0.3
Day 2	6.3^a ^± 2.5	1.2 ± 0.4	23.4^a ^± 2.0	1.5 ± 0.5	0.4 ± 0.3	1.3 ± 0.7	3.7^a ^± 0.6	1.1 ± 0.3	157.5^a ^± 21.2	1.2 ± 0.3
Day 3	7.2^a ^± 2.7	1.1 ± 0.2	11.0* ± 2.7	1.1 ± 0.2	0.7 ± 0.5	1.1 ± 0.6	2.0 ± 0.4	1.0 ± 0.2	58.2^a ^± 6.4	1.4 ± 0.6
Day 7	3.3^a ^± 0.7	1.0 ± 0.2	5.9 ± 2.3	1.1 ± 0.2	0.3 ± 0.2	1.2 ± 0.6	0.7 ± 0.3	1.0 ± 0.2	79.7^a ^± 42.2	1.1 ± 0.3
Day 14	3.2^a ^± 1.4	1.1 ± 0.2	2.3 ± 0.3	1.0 ± 0.1	0.8 ± 0.5	1.1 ± 0.5	0.7 ± 0.2	1.0 ± 0.1	28.7^a ^± 7.1	1.3 ± 0.5

Rats were immunized subcutaneously with 0.5 g of methylated bovine serum albumin (mBSA) emulsified with complete Freund's adjuvant 21 and 14 days prior to articular injection (day 0) of 0.5 mg of mBSA (sensitized) or 50 μL of saline (contralateral) into the knee joints. Expression of mRNA was assessed before (on day 0) and after (at 7 hours and on days 1, 2, 3, 7, and 14) sensitization. Data are expressed as delta delta threshold cycle (Ct) related to the housekeeping gene (*RP29*). Values are the mean ± standard error of the mean of five independent measurements per time. ^a^*P *< 0.05 in comparison with day 0. IL, interleukin; MCP-1, monocyte chemoattractant protein-1; TNF-α, tumor necrosis factor-alpha; VEGF, vascular endothelial cell growth factor.

### Overall pattern of mediator levels in biological fluids

**Figure 2:**
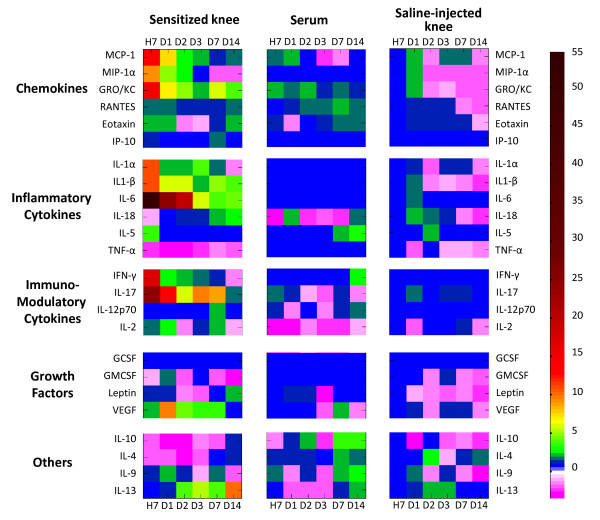
**Overall profiling of cytokines in joints and bloodstream of rats developing antigen-induced arthritis**. Synovial fluid of both knees - sensitized with methylated bovine serum albumin (mBSA) and saline-injected - and sera were collected at necropsy, and mediator levels were measured by multiplex immunoassay. Results are expressed as fold of induction or of inhibition in comparison with day of sensitization (day 0) (*n *= 5, representative of three independent experiments). Blue squares indicate that the corresponding mediator was at the basal level, dark red squares indicate the maximal induction fold (50×), and purple squares indicate the maximal inhibition level (/5). D, day; G-CSF, granulocyte colony-stimulating factor; GM-CSF, granulocyte macrophage colony-stimulating factor; GRO/KC, growth-related oncogene/keratinocyte chemoattractant; H, hour; IFNγ, interferon gamma; IL, interleukin; IP-10, inducible protein-10; MCP-1, monocyte chemoattractant protein-1; MIP-1α, macrophage inflammatory protein-1-alpha; RANTES, regulated on activation normal T expressed and secreted; TNF-α, tumor necrosis factor-alpha; VEGF, vascular endothelial cell growth factor.

As shown in Figure 2, the global analysis of arthritis-induced changes in cytokine amounts were more marked in SF than in the bloodstream, regardless of the functional class considered. Mediators displayed two main profiles of variation in arthritic joints, and cytokines showed either an early and transient increase or a delayed and sustained increase in their synovial contents. In general, cytokine amounts increased earlier and more importantly in arthritic knees than in contralateral saline-injected knees, although some mediators, such as IP-10, IL-12p70, TNFα, or IL-5, failed to be affected significantly by the arthritic process. A similar lack of change was noted for the growth factors G-CSF and GM-CSF in all biological fluids, whereas a marginal decrease of IL-4 and IL-10 levels was observed.

### Cytokines with an early release in arthritic fluid

**Figure 3:**
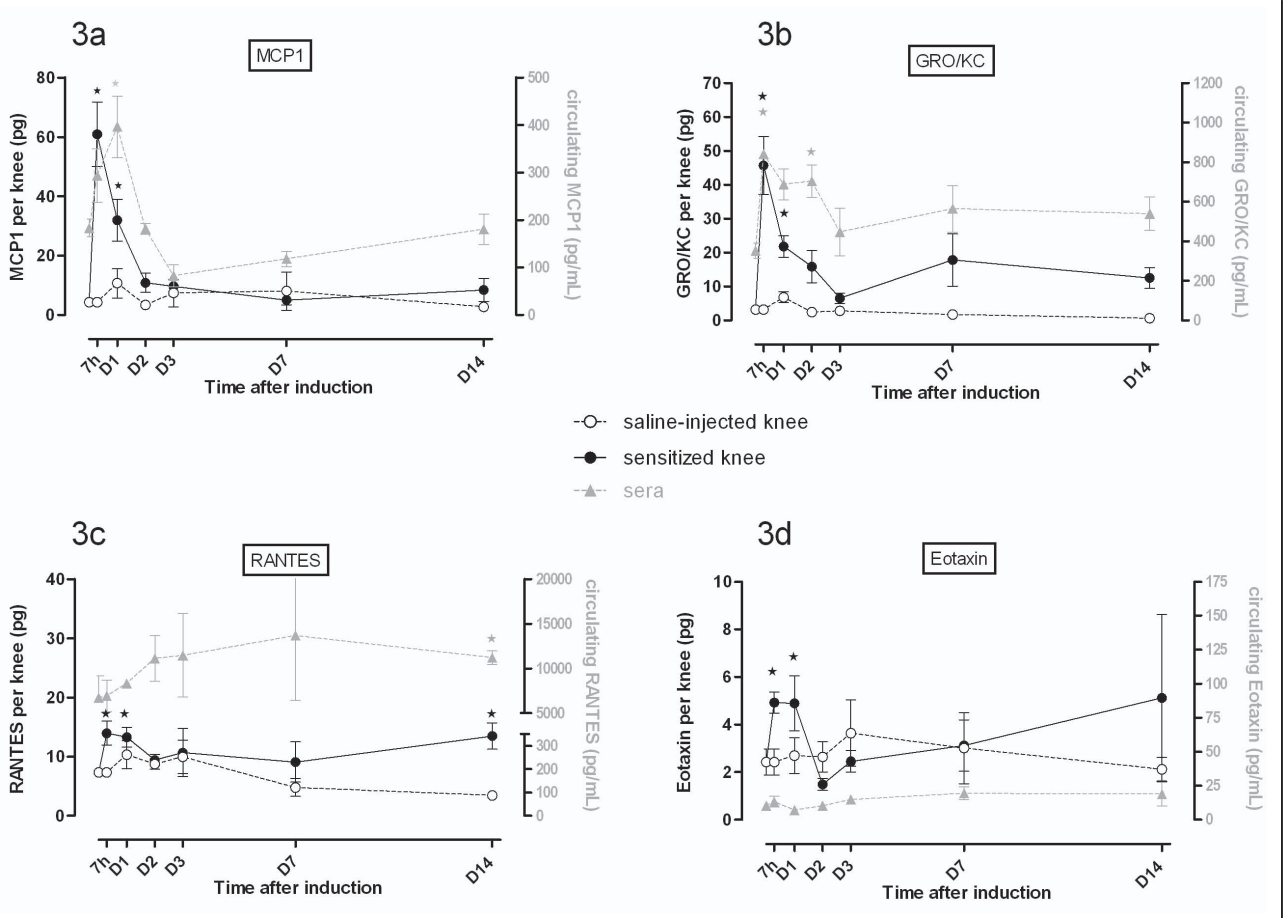
**Synovial and circulating profiles of chemokines displaying an early increase in their joint levels during antigen-induced arthritis**. Expression of protein was assessed before the induction of antigen-induced arthritis (day 0) or afterwards (at 7 hours and on days 1, 2, 3, 7, and 14) in both arthritic (mBSA-sensitized) and contralateral (saline-injected) knees and in sera. Results are expressed as concentrations (in picograms per milliliter) in serum and as quantity by knee (in picograms per knee) of the chemokines monocyte chemoattractant protein-1 (MCP-1) **(a)**, growth-related oncogene/keratinocyte chemoattractant (GRO/KC) **(b)**, regulated on activation normal T expressed and secreted (RANTES) **(c)**, or eotaxin **(d)**. Values are the mean ± standard error of the mean of five independent samples. *P < 0.05 in comparison with day 0. D, day; h, hour; mBSA, methylated bovine serum albumin.

The amounts of the chemokines MIP-1α, MCP-1, GRO/KC, eotaxin, and RANTES increased by 9-, 14-, 14-, 2-, and 2-fold, respectively, in the SF of arthritic rats within 7 hours after the antigenic challenge (Figure 2). No significant changes were observed in the SF of the contralateral knee. In sera, GRO/KC, MCP-1, and RANTES levels were also increased, although the range of induction remained limited (2.7-, 2-, and 1.8-fold, respectively) since these chemokines displayed a high circulating level under basal conditions (Figure 3a,b,c). The intra-articular peaks of mediator averaged 60 pg/knee for MCP-1 (Figure 3a), 45 pg/knee for GRO/KC (Figure 3b), 15 pg/knee for RANTES (Figure 3c), and 5 pg/knee for eotaxin (Figure 3d). These chemokines returned to basal levels in arthritic fluids and sera within D_3_, but a secondary flare of RANTES release (2-fold change) was observed on D_14_.

**Figure 4:**
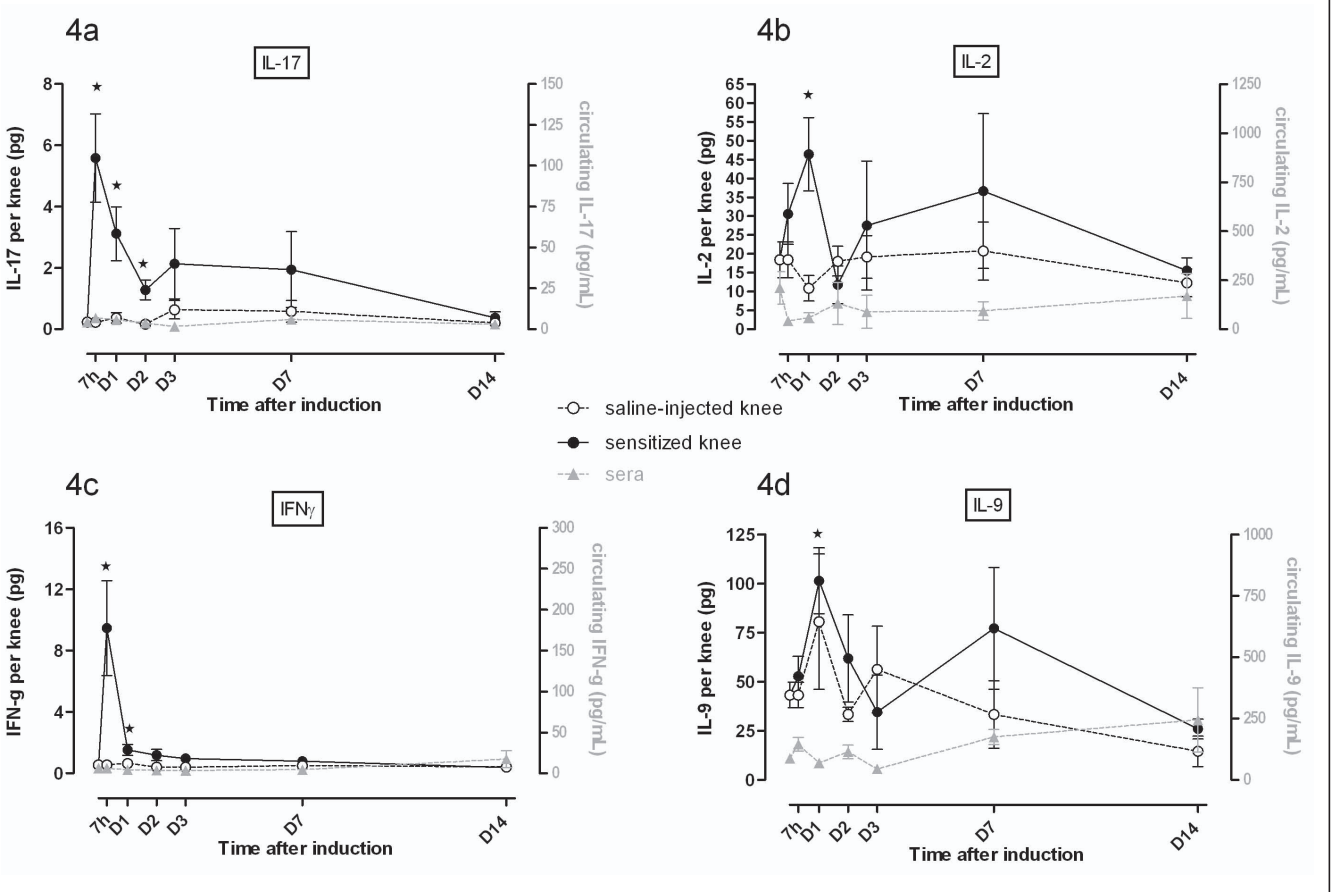
**Synovial and circulating profiles of immunomodulatory cytokines displaying an early increase in their joint levels during antigen-induced arthritis**. Expression of protein was assessed before the induction of antigen-induced arthritis (day 0) or afterwards (at 7 hours and on days 1, 2, 3, 7, and 14) in both arthritic (mBSA-sensitized) and contralateral (saline-injected) knees and in sera. Results are expressed as concentrations (in picograms per milliliter) in serum and as quantity by knee (in picograms per knee) of the immunomodulatory cytokines interleukin-17 (IL-17) **(a)**, IL-2 **(b)**, and interferon-gamma (IFNγ) **(c) **and IL-9 **(d)**. Values are the mean ± standard error of the mean of five independent samples. *P < 0.05 in comparison with day 0. D, day; h, hour; mBSA, methylated bovine serum albumin.

The immunomodulatory cytokines IL-17 and IFNγ increased by 25- and 21-fold, respectively, in the SF of arthritic rats within 7 hours after the antigenic challenge (Figure 4a,c). However, both cytokines displayed a different time course since IFNγ returned to basal levels from D_1 _after mBSA injection, whereas IL-17 levels declined gradually until D_14_. No changes in cytokine amounts were observed in the joint fluid from saline-injected knees or in sera. IL-2 and IL-9 contents increased less rapidly than IL-17 and IFNγ in the arthritic knee (2- to 2.5-fold induction by D_1_) and showed a secondary but non-significant increase by D_7 _(Figure 4b,d).

### Cytokines with a sustained or a late release in arthritic fluid

**Figure 5:**
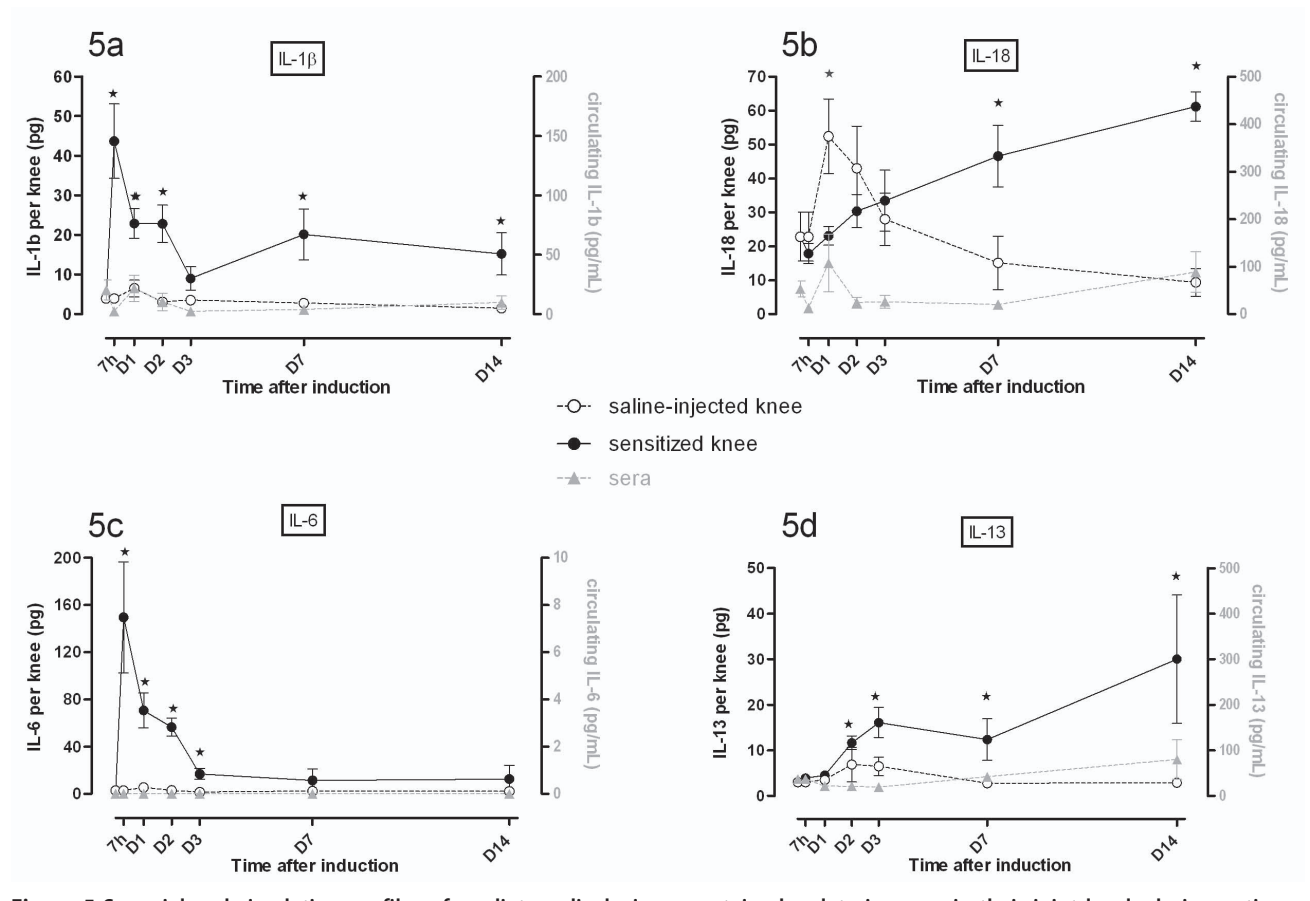
**Synovial and circulating profiles of mediators displaying a sustained or late increase in their joint levels during antigen-induced arthritis**. Expression of protein was assessed before the induction of antigen-induced arthritis (day 0) or afterwards (at 7 hours and on days 1, 2, 3, 7, and 14) in both arthritic (mBSA-injected) and contralateral (saline-injected) knees and in sera. Results are expressed as concentrations (in picograms per milliliter) in serum and as quantity by knee (in picograms per knee) of the proinflammatory cytokines interleukin-1-beta (IL-1β) **(a)**, IL-18 **(b)**, or IL-6 **(c) **and the T helper 2 cytokine IL-13 **(d)**. Values are the mean ± standard error of the mean of five independent samples. *P < 0.05 in comparison with day 0. D, day; h, hour; mBSA, methylated bovine serum albumin.

As early as 7 hours after arthritis induction, the amounts of the proinflammatory cytokines IL-1β and IL-6 increased by 12- and 55-fold, respectively, in the SF of sensitized joints (Figure 5a,c). However, IL-1 isoforms displayed a distinct pattern of evolution since IL-1β levels remained significantly elevated in arthritic joints until D_14 _(Figure 5a) whereas IL-1α declined progressively (Figure 2). The intra-articular peaks of mediators averaged 150 pg/knee for IL-6 and 45 pg/knee for IL-1β (Figure 5a,c), but no significant variation was noted in corresponding sera. In arthritic fluid, the level of IL-18 and IL-13 increased only from D_2 _and D_7 _(2-fold increase) until D_14 _(3- and 4-fold increases), respectively (Figure 5b,d). However, an unexpected 2.2-fold increase in IL-18 level was observed in the contralateral knee from D_1 _to D_2 _after the antigenic challenge (Figure 5b). Such variation was consistent with the transient release of IL-18 into the bloodstream by D_1_.

### Expression profile of selected growth factors

A delayed and sustained release of VEGF was observed in the sensitized knees, and a 10-fold increase by D_1 _was followed by a progressive lessening until D_14 _(Figure 2). Despite its high circulating levels, leptin showed an inverse evolution profile in arthritic SF but was significantly increased by D_14 _in comparison with contralateral knees.

### Histological examination

**Figure 6:**
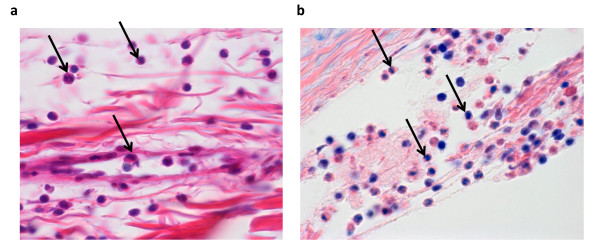
**Eosinophil infiltration in the synovium of rats developing antigen-induced arthritis**. Representative sections of arthritic knee (mBSA-injected) 7 hours after arthritis induction are shown. **(a) **Hematoxylin/eosin/safran staining (magnification 100×). **(b) **May-Grünwald Giemsa (magnification 63×). Eosinophils are indicated by arrows. mBSA, methylated bovine serum albumin.

**Figure 7:**
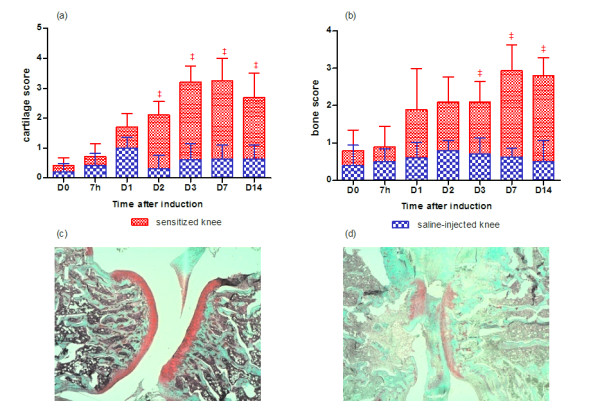
**Histological grading of joint lesions in the knee joint of rats developing antigen-induced arthritis**. **(a) **Cartilage score. **(b) **Bone score. Data are expressed as mean ± standard error of the mean of five independent samples. ^‡^*P *< 0.05 in comparison with day 0. Representative sections of contralateral (saline-injected) **(c) **and arthritic (mBSA-injected) **(d) **knees 14 days after arthritis induction (safranin O-fast green staining, magnification 4×). D, day; h, hour; mBSA, methylated bovine serum albumin.

As early as 7 hours after arthritis induction, synovium was infiltrated by migrating cells, especially in the perivascular areas of the sensitized knees (Table 2). A more dedicated staining (May-Grünwald Giemsa) of blood cells indicated infiltration by eosinophils in the knee as early as 7 hours after arthritis induction (Figure 6). Interestingly, though maximal at the earliest time point, the perivascular infiltration paralleled the time course of the diffuse infiltration of the tissue with a progressive lessening until D_14_. The only exception was the transient infiltration that was detected around the blood vessels before D_2 _in the contralateral knees (Table 2). No other histological finding was detected in contralateral knees throughout disease duration. In sensitized knees, the additional pathological changes were a progressive thickening of the synovial cell layer from D_1 _to D_14 _and an overall tissue fibrosis. Interestingly, new blood vessel formation occurred at a later time point (D_3 _to D_7_) in the inflamed rat synovium. The scoring of cartilage lesions after safranin O staining (Figure 7a) revealed a progressive loss of proteoglycan in the sensitized knees (Figure 7c,d) and the loss became significant from D_2 _to D_14_. A concomitant bone loss was observed (Figure 7b,d) in comparison with saline-injected knees (Figure 7b,c).

**Table 2:** Histological grading of knee synovium in rats developing antigen-induced arthritis

Time point	Synovial lining hyperplasia		Fibrosis		Proliferative blood vessels		Diffuse infiltrates		Perivascular infiltrates	
	Sensitized	Saline-injected	Sensitized	Saline-injected	Sensitized	Saline-injected	Sensitized	Saline-injected	Sensitized	Saline-injected
Day 0	0.2 ± 0.2	0.2 ± 0.2	0.6 ± 0.2	0.6 ± 0.3	0.6 ± 0.2	0.6 ± 0.3	0.4 ± 0.3	0.4 ± 0.3	0.0 ± 0.0	0.0 ± 0.0
7 hours	1.2 ± 0.4	0.2 ± 0.2	0.8 ± 0.4	0.6 ± 0.3	0.8 ± 0.2	0.8 ± 0.2	1.2^a ^± 0.2	0.8 ± 0.4	2.8^a ^± 0.6	1.6^a ^± 0.6
Day 1	1.6^a ^± 0.4	0.8 ± 0.2	1.6^a ^± 0.4	0.6 ± 0.3	0.6 ± 0.2	0.4 ± 0.2	2.2^a ^± 0.4	1.0 ± 0.0	2.0^a ^± 0.5	1.8^a ^± 0.4
Day 2	2^a ^± 0.3	0.2 ± 0.2	2.4^a ^± 0.3	0.6 ± 0.3	1.0 ± 0.0	0.6 ± 0.4	3.4^a ^± 0.3	0.4 ± 0.3	2.6^a ^± 0.5	0.4 ± 0.3
Day 3	3.2^a ^± 0.6	0.4 ± 0.3	2.2^a ^± 0.5	0.6 ± 0.3	1.8^a ^± 0.2	0.8 ± 0.4	3.4^a ^± 0.4	0.4 ± 0.3	2.0^a ^± 0.5	0.4 ± 0.3
Day 7	2.5^a ^± 0.7	0.3 ± 0.3	3.5^a ^± 0.3	0.3 ± 0.3	2.5^a ^± 0.5	0.5 ± 0.3	2.8^a ^± 0.6	0.3 ± 0.4	1.8^a ^± 0.5	0.4 ± 0.4
Day 14	3.3^a ^± 0.5	0.0 ± 0.0	4.0^a ^± 0.0	0.3 ± 0.3	1.8 ± 0.5	1.0 ± 0.4	2.0^a ^± 0.0	0.3 ± 0.3	1.8^a ^± 0.3	0.8 ± 0.3

Rats were immunized subcutaneously with 0.5 g of methylated bovine serum albumin (mBSA) emulsified with complete Freund's adjuvant 21 and 14 days prior to articular injection (day 0) of 0.5 mg of mBSA (sensitized) or 50 μL of saline (contralateral) into the knee joints. Values are expressed as the mean ± standard error of the mean of five rats per time. ^a^*P *< 0.05 in comparison with day 0.

## Discussion

The major aim of the present study was to analyze the relevance of cytokine profiling to the pathophysiology of experimental arthritis by combining the originality of a sampling method of SF in rodents and the screening capacity of a multiplex-based system. Indeed, despite the classical use of the AIA model, the kinetics of mRNA and mediator expression in arthritic joints remain poorly characterized, whereas very few face-off studies have been carried out between articular and circulating mediator levels.

During the course of AIA, we confirmed that joint swelling occurred early and peaked by D_2_, when the incapacitation of the animal became maximal. Articular incapacitation is assumed to result from the altered nociception following injection of an inflammatory trigger into the joints [29]. Although there can be some differences between the use of zymosan, carrageenan, or LPS as arthritis inducers, knee joint incapacitation is generally thought to be supported by the release of bradykinin, nitric oxide, eicosanoids, and/or proinflammatory cytokines [30]. We describe - to our knowledge, for the first time - a long-lasting joint incapacitation after a local antigenic challenge. The best correlation with the fast occurrence of abnormal weight bearing until D_2 _was found with IL-2 (r_s _= 1) over VEGF, although the early peak release of the chemokines MCP-1, MIP-1α, and GRO/KC and the proinflammatory cytokines IL-1α or -β, IL-17, or IFNγ may have contributed to the initiation of incapacitation. Nevertheless, at later time points, joint swelling lessened progressively whereas abnormal weight bearing remained at the same level, suggesting that pain persisted despite the partial resolution of inflammation, likely as a result of severe joint tissue remodeling. Because of its prominently local character, AIA allows the analysis of cytokine patterns in a locally driven immune response. We showed that arthritis was characterized by an early transcriptional activation of the chemokine MCP-1, the proinflammatory cytokines IL-1β, IL-6, and TNFα, and the angiogenic factor VEGF in synovial tissue. For most cytokines, this early induction of mRNA expression in the inflamed joint was consistent with the early release of corresponding proteins in the arthritic fluid. However, the transcriptional regulation cannot be considered the unique mechanism responsible for increased cytokine levels in physiological fluid, since the release of proteins, notably chemokines, appeared as early as 7 hours, suggesting the existence of pools of mRNA or cytokine precursors (or both) which were prone to be processed. In addition, the extent of cytokine expression was much lower at the mediator than the transcript level, and the release of IL-1β and IL-6 preceded the maximal increase in their mRNA levels, supporting the possible contribution of rate-limiting steps between both processes. One cannot rule out that very early increase in mRNA may have preceded a rapid decline due to mRNA instability or action of miRNA, as has been suggested for IL-1β [31]. A similar observation has been made for IL-6 in the murine air pouch model that resembles the human synovial membrane. Indeed, injection of monosodium urate in the pouch induced IL-6 expression in the first hour after challenge and before a drastic decrease to basal levels between 4 and 9 hours [32]. Therefore, we cannot rule out a transient and precocious induction of IL-1β and IL-6 mRNA in our experimental conditions.

As a consequence, the contribution of a given mediator to arthritis could be overestimated when extrapolated only from its mRNA level in inflamed joint tissues [20,21]. However, we observed a surprising uncoupling between the early overexpression of TNFα in the inflamed synovium and its stable low amount in the arthritic fluid, despite previous reports of a lack of correlation between mRNA and protein expression [21]. As TNFα-converting enzyme is thought to be activated in rats developing arthritis [33], especially in the early stage of the disease [34], the low joint TNFα levels are unlikely to reflect a defective processing of its mRNA. We suggest that these low levels can reflect either a fast degradation of the cytokine by proteases or the inability of the multiplex immunoassay to recognize TNFα when combined with soluble receptors able to mask protein recognition by bead antibodies. In line with such possible interference, protein arrays were reported to be unable to detect IL-1α [35] or TNFα [36] in culture supernatants, despite their use as the cell challenging inflammatory stimulus. In experimental arthritis, a pattern of soluble proteins is much more relevant than a pattern of transcripts to pathological mechanisms because most cytokines exert their biological effects in an endocrine, paracrine, or juxtacrine way. From a practical point of view, blood sampling is easy to perform and circulating levels of mediators have been shown to predict disease onset in humans [37] or to be indicative of a patient's response to biologics [38,39], especially when taking advantage of a multiplex analysis [15]. Some correlations with disease activity were also reported in RA [40] or experimental arthritis [41]. However, other studies support the view that the local expression of cytokines may be the best predictor of joint damage progression in humans [19] and that blood levels do not necessarily correlate to clinical response in animal models [21]. In addition, a multiplex analysis of SF demonstrated that the follow-up of cytokine pattern allowed patients who had early RA and subsequently developed RA to be distinguished from those who had early RA but did not develop RA [42]. When we performed a comparative kinetic study of mediator levels in serum and SF, we confirmed that the expression pattern was much more informative in joint fluid than in blood [43]. Indeed, serum concentrations showed only little variation during AIA, whereas some correlations were established between cytokine concentrations in arthritic SF and histological or clinical parameters. Such results are consistent with the local and monoarticular nature of AIA [44], rendering the amount of cytokines produced within a single diseased joint prone to be reduced by degradation in the lymphatic system or by dilution into the bloodstream or both [45].

Chemokines were the first mediators to be overexpressed in biological fluids. However, MCP-1, GRO/KC, and RANTES levels increased simultaneously in arthritic SF and serum whereas MIP-1α and eotaxin were induced only in sensitized knees during the first 24 hours of arthritis. Chemokines play a key role in inflammation by recruiting mononuclear leukocytes and lymphocytes in inflamed knee joints [3,46,47]. Their expression was clearly correlated to perivascular and diffuse infiltrates in the synovial membranes of sensitized knees, in agreement with their chemotactic potential [46]. Eotaxin is a chemokine that is implicated in allergic responses [48] by recruiting eosinophils. Its involvement in arthritic pathologies has not yet been demonstrated [49], but multiplex analysis of plasma of patients with RA showed elevated levels of eotaxin versus healthy controls [37]. Furthermore, the expression profile of eotaxin is very similar to that of RANTES in inflamed SF. Previous studies showed that RANTES played a fundamental role in the trafficking and activation of leukocytes in arthritis establishment [46,50] and that the use of RANTES antagonists could lead to reduced joint inflammation [51,52]. We can speculate that eotaxin could be a potential target in RA treatment. Eotaxin-2 is a potent chemoattractant for eosinophils, basophils, and Th2 lymphocytes. Here, we demonstrated the presence of eosinophils in arthritic joints in the first hours of arthritis development, whereas eosinophils were reported to be absent from the synovium ofpatients with late-stage arthritis [53]. But in their observations, Tetlow and Woolley [53] did not exclude the possibility that eosinophils show a transient and rapid infiltration of rheumatoid synovial tissue at earlier stages of disease activity. Furthermore, RA is considered a heterogeneous disease, as treatments targeting different pathogenic pathways such as neutralization of TNF-α or depletion of B cells are not effective in all patients. It is likely that patients with resistant RA may have other types of disease, and one uncommon type of RA is associated with hypereosinophilia, which correlates to arthritis severity [54]. GRO/KC is a CXC chemokine whose pathogenic role is poorly described in arthropathies but displayed an interesting expression profile in the AIA model. MCP-1, RANTES, and MIP-1α are produced in inflamed knees [46,55,56] and act in the recruitment of phagocytic leukocytes during inflammation. Globally, chemokine induction in SF is positively correlated with joint swelling in the early phase of arthritis and synovium infiltration observed at histological examination.

Proinflammatory cytokines also increased early in SF of arthritic knees. IL-1α and IL-1β are expressed very precociously and can be linked to the proteoglycan anabolism loss observed in patellar cartilage, and IL-1β has a key role in the regulation of proteoglycan metabolism [11]. Biotherapies targeting IL-1β have already shown their efficacy in inflammation healing [10] by using either antibodies or antagonist receptor (IL-1Ra). Bone remodeling was observed and was correlated to IL-1β [9,22] and IL-6 expression patterns. IL-6 was also expressed early and at very high levels in SF and remained elevated during the course of this model. IL-6 is now considered as one of the most important mediators of the acute-phase response. As previously discussed, we failed to observe any significant modifications of TNF-α level during the course of AIA; nevertheless, its pivotal role in AIA establishment was demonstrated by the efficacy of anti-TNF-α in this model [57]. Previous studies showed a maximum of induction 6 hours after arthritis induction but this measure was performed on synovial membrane homogenates, and consequently the measured TNF-α level addressed both transmembrane and extracellular mediators. IL-1β, IL-6, and TNFα are elevated in human RA synovium and have also been shown to contribute to the development of arthritis in animal models [4,58].

IL-18 belongs to the IL-1 family and is processed like IL-1β by the inflammasome [59] but presented a different expression profile in SF. Surprisingly, we observed an increase in the contralateral knee at 7 hours. Because of the prominently local character of AIA, it is commonly accepted that each rat could be its own control, since only the antigen-injected joint develops inflammation. Nevertheless, this observation points out that the contralateral knee may favor misinterpretation of the data when considered as the healthy control [57].

In sensitized knees, IL-18 level was progressively increased. IL-18 is thought to play a major role in the chronicity of inflammation in this arthritis model. Recent publications have demonstrated the importance of this cytokine in allergies [60] and arthritic pathologies [10]. Although IL-18 is also known to act synergistically with IL-12, we did not observe correlations between these two cytokines in SF during AIA. IL-18 was also known as an IFNγ-stimulating factor [61], but we observed two distinct expression profiles for IFNγ and IL-18 in this study. Immunomodulatory cytokines play a major role in RA. Indeed, in this experimental model, we observed that IFNγ and IL-2 were expressed in SF, and only in the first hours after sensitization.

Until recently, arthritis was considered a Th1 pathology, but now arthritis is known to be mainly IL-17-dependent [62]. Many studies are actually conducted to target IL-17 in arthritis models [62,63] because of its potential synergistic effects with IL-1β, IL-6, and TNF-α in inducing cytokine expression and joint damage [64]. The expression profile of IL-17 was the same as that of IL-1β, and IL-17 cytokine may also contribute to inhibition of proteoglycan anabolism [65].

In regard to growth factors, VEGF was expressed after 24 hours and the histological analysis revealed an increased number of blood vessels in the synovium after 4 days, consistently with the promotion of neoangiogenesis by this cytokine [66]. Levels of G-CSF and GM-CSF did not show significant variations, and their importance seems limited in this experimental arthritis model, even if their antagonists have shown anti-inflammatory effects in other murine experimental models [67,68]. Sera leptin decreased significantly after 3 days and then returned to its original high level, whereas in SF, an increase was observed after 14 days, indicating the potential role of this adipokine in arthritis chronicity and bone degradation in the long term. There is a consensus about its pivotal role as a proinflammatory, bone metabolism regulating, and immune-modulating agent. Nevertheless, its mechanisms of action have not been fully described [69].

We observed, in SF, a progressive increase in the amount of IL-13, a cytokine usually described in allergic reactions and pulmonary fibrosis [70]. However, the pattern of IL-4 was different from that of IL-13. Although IL-13 and IL-4 are both central Th2 cytokines in the immune system and potent activators of inflammatory responses and fibrosis during Th2 inflammation, recent studies demonstrated that these cytokines exerted a distinct role in asthma pathology [71]. One can suppose that IL-13 rather than IL-4 plays a prominent role in the synovial membrane fibrosis observed here since a positive chronic correlation exists between IL-13 and fibrosis (Spearman correlation). Another cytokine not described in arthritis but known for its cell proliferation capacities [72], IL-9, was expressed early in sensitized knees. Both cytokines may play a role in synovial hyperplasia, which is a major event in RA. Further studies will be required to confirm this hypothesis.

## Conclusions

The present study is the first kinetic study (from 7 hours to 14 days) of 24 SF cytokines profiled in a complete biochemical, histological, and clinical arthritis model. Previous studies on animal models focused on a restricted number of cytokines [21] and were assessed on synovial extract or joint homogenates (which contain both inactive cytokine precursors and active cytokines) [20]. Using this technology, we observed that SF cytokine amounts are correlated to biochemical, histological, and clinical parameters of arthritis in a timely fashion. Moreover, we established expression patterns of cytokines - besides the mediators known to be involved in arthritis - that are poorly described in articular physiopathology. The analysis provided here may allow the further study of molecular mechanisms involved in the establishment and evolution of autoimmune arthritis, leading to potential new clinical therapies [73].

## Abbreviations

AIA: antigen-induced arthritis; D: day; G-CSF: granulocyte colony-stimulating factor; GM-CSF: granulocyte macrophage colony-stimulating factor; GRO/KC: growth-related oncogene/keratinocyte chemoattractant; IFNγ: interferon gamma; IL: interleukin; IP-10: inducible protein-10; mBSA: methylated bovine serum albumin; MCP-1: monocyte chemoattractant protein-1; MIP-1α: macrophage inflammatory protein-1-alpha; PBS: phosphate-buffered saline; PCR: polymerase chain reaction; RA: rheumatoid arthritis; RANTES: regulated on activation normal T expressed and secreted; SF: synovial fluid; Th: T helper; TNF: tumor necrosis factor; VEGF: vascular endothelial cell growth factor.

## Competing interests

The authors declare that they have no competing interests.

## Authors' contributions

JP helped to perform all *in vivo *and molecular studies. DM helped to perform all *in vivo *and molecular studies and to supervise the study design and the manuscript, conceived the study, and participated in its design and final presentation. J-CG and LG helped to perform molecular and histological studies and statistical analysis. AP and CC-H helped to perform molecular and histological studies. CD performed histological analysis. PG, PN helped to supervise the study design. J-YJ helped to supervise the study design and the manuscript and final presentation. All authors read and approved the final manuscript.
